# Zirconium and Yttrium Co-Doped BaCo_0.8_Zr_0.1_Y_0.1_O_3−δ_: A New Mixed-Conducting Perovskite Oxide-Based Membrane for Efficient and Stable Oxygen Permeation

**DOI:** 10.3390/membranes12090831

**Published:** 2022-08-25

**Authors:** Zixiang Xu, Jian Yu, Wei Wang

**Affiliations:** State Key Laboratory of Materials-Oriented Chemical Engineering, College of Chemical Engineering, Nanjing Tech University, Nanjing 210009, China

**Keywords:** oxygen permeation, perovskite oxides, co-doping, structural stability, BaCoO_3−δ_

## Abstract

Oxygen permeation membranes (OPMs) are regarded as promising technology for pure oxygen production. Among various materials for OPMs, perovskite oxides with mixed electron and oxygen-ion (e^−^/O^2−^) conducting capability have attracted particular interest because of the high O^2−^ conductivity and structural/compositional flexibility. However, BaCoO_3−δ_-based perovskites as one of the most investigated OPMs suffer from low oxygen permeation rate and inferior structural stability in CO_2_-containing atmospheres. Herein, zirconium and yttrium co-doped BaCoO_3−δ_ (BaCo_1−2x_Zr_x_Y_x_O_3−δ_, x = 0, 0.05, 0.1 and 0.15) are designed and developed for efficient and stable OPMs by stabilizing the crystal structure of BaCoO_3−δ_. With the increased Zr/Y co-doping content, the crystal structural stability of doped BaCoO_3−δ_ is much improved although the oxygen permeation flux is slightly reduced. After optimizing the co-doping amount, BaCo_0.8_Zr_0.1_Y_0.1_O_3−δ_ displays both a high rate and superior durability for oxygen permeation due to the well-balanced grain size, oxygen-ion mobility, crystal structural stability, oxygen vacancy concentration and surface exchange/bulk diffusion capability. Consequently, the BaCo_0.8_Zr_0.1_Y_0.1_O_3−δ_ membrane delivers a high oxygen permeation rate of 1.3 mL min^−1^ cm^−2^ and relatively stable operation at 800 °C for 100 h. This work presents a promising co-doping strategy to boost the performance of perovskite-based OPMs, which can promote the industrial application of OPM technology.

## 1. Introduction

Perovskite oxides with electron and oxygen-ion (e^−^/O^2−^) conducting capability have been extensively investigated in oxygen permeation membranes (OPMs), electrodes for ceramic fuel cells, catalysts for photo(electro)chemical water splitting and advanced oxidation processes due to the compositional/structural flexibility, tunable physical/chemical properties and high chemical/thermal stability [1,2,3,4,5,6,7,8,9,10,11,12]. Oxygen separation by using perovskite oxide-based membranes exhibited several advantages such as high selectivity and low cost over traditional pressure swing adsorption and low-temperature air separation [13,14,15]. Although numerous perovskite materials have been designed and developed as OPMs, the oxygen permeability and durability are still insufficient for practical applications. In addition, perovskite-based membranes should display superior structural and chemical stability under oxygen permeation conditions including high temperature, CO_2_-containing atmosphere and large differences in oxygen partial pressures at two sides of OPMs [16,17,18]. However, the materials developed at present cannot fulfill the complex prerequisites of the large-scale oxygen permeation application. Thus, it is still of great significance to design new materials for efficient and stable oxygen permeation.

In past decades, perovskite oxides have received increasing interest as high-performance OPM materials due to the abundant compositional elements and easy adjustment in the structure and chemical/physical properties [19,20]. Among them, cobalt (Co)-based perovskite membranes have attracted particular attention because of the superb oxygen permeation rate [21,22,23,24,25]. For instance, Teraoka et al. have firstly investigated the performance of La_1−x_Sr_x_Co_1−y_Fe_y_O_3−δ_ OPMs and they found that SrCo_0.8_Fe_0.2_O_3−δ_ (SCF) displayed a superior oxygen permeation rate to other investigated OPMs due to the larger oxygen vacancy concentration [26]. Nevertheless, SCF suffered from a serious crystal structure transition from cubic to brownmillerite under lower oxygen partial pressures and reduced temperatures (<800 °C), leading to a much-decreased oxygen permeability and durability [27]. To stabilize the crystal structure of SCF under oxygen permeation conditions, selectively doping at the A-site (e.g., Ba^2+^, La^3+^) and/or B-site (e.g., Nb^5+^, Ti^4+^) of SCF are reported to be effective to improve the crystal structure stability and/or oxygen permeation performance of SCF [28,29,30,31]. Caro et al. also studied the influences of Co/Fe ratios on the oxygen permeability of Pr-doped SCF for OPMs and Pr_0.6_Sr_0.4_Co_0.2_Fe_0.8_O_3−δ_ perovskite showed a durable phase structure and a relatively high oxygen flux among various Pr-doped SCF perovskites [32]. In another study, Jin et al. have studied the effects of Zr^4+^ doping amounts on the oxygen permeation performance and phase stability of SCF and the Zr^4+^ doping greatly improved the stability of SCF for OPMs [33].

BaCoO_3−δ_ has been widely employed as a parent oxide for the design of numerous perovskite oxides for OPMs, showing relatively high oxygen permeability due to the superior oxygen surface exchange capability, large oxygen vacancy amount and free lattice volume, etc. [34,35]. However, an ideal perovskite structure cannot be formed in pure BaCoO_3−δ_ because of the large differences in the ionic sizes of Ba^2+^ and Co^x+^, leading to a highly distorted structure, which may block the bulk diffusion of O^2−^ and electron transfer [36]. The doping strategy has been widely employed to design and develop BaCoO_3−δ_-based perovskite oxides for OPMs with improved oxygen permeation rate and durability by tuning the phase structure, oxygen vacancy amount, phase stability, oxygen mobility, oxygen surface exchange properties, etc. [37,38]. As one of the typically doped perovskite oxides for OPMs, Ba_0.5_Sr_0.5_Co_0.8_Fe_0.2_O_3−δ_ exhibited a cubic structure and a superior oxygen permeation rate [39]. Herein, two stable cations with relatively low valences (e.g., Zr^4+^, Y^3+^) are co-doped into the B-site of BaCoO_3−δ_ to enhance the oxygen permeation rate/durability and stabilize the phase structure. Y dopant is chosen to improve the structural stability of the perovskite-based OPMs, which also acts as a sintering aid to promote the densification of the membranes while Zr is selected to further improve the phase stability and the CO_2_ resistance of the perovskite-based OPMs [40,41]. With the increased Zr/Y co-doping concentrations in BaCo_1−2x_Zr_x_Y_x_O_3−δ_ (BCZY, x = 0, 0.05, 0.1 and 0.15), the phase transition from hexagonal (x = 0, BC) to cubic (x = 0.05, 0.1 and 0.15) was observed. The optimized BaCo_0.8_Zr_0.1_Y_0.1_O_3−δ_ perovskite displayed a relatively high rate and durability for oxygen permeation due to the well-balanced particle sizes, O^2−^ conductivity, crystal structural stability, oxygen vacancy amount as well as bulk diffusion and surface exchange capability of oxygen species. As a result, the BaCo_0.8_Zr_0.1_Y_0.1_O_3−δ_ membrane displayed a superior oxygen permeation rate of 1.3 mL min^−1^ cm^−2^ and a relatively high oxygen permeation stability for 100 h at 800 °C. This study presents a highly promising mixed-conducting perovskite material for efficient and durable OPMs, promoting the industrialization of this technology.

## 2. Materials and Methods

### 2.1. Materials and Membrane Synthesis

Various BaCo_1−2x_Zr_x_Y_x_O_3−δ_ (BCZY, x = 0, 0.05, 0.1 and 0.15) powders including BaCoO_3_, BaCo_0.9_Zr_0.05_Y_0.05_O_3−δ_ (BCZY1), BaCo_0.8_Zr_0.1_Y_0.1_O_3−δ_ (BCZY2) and BaCo_0.7_Zr_0.15_Y_0.15_O_3−δ_ (BCZY3) were synthesized by a sol-gel route [42]. The obtained BCZY powders were then mixed in ethanol media by ball milling (Fritsch, Pulverisett 6) for 30 min to obtain fine and homogeneous powders. Dense BCZY membranes (15 mm in diameter) were fabricated by dry-pressing and further annealed at 1100 °C in air for 10 h [43]. The as-synthesized OPMs were further polished by 1500-mesh SiC sandpaper on both sides to achieve various thicknesses of 0.6, 0.8 and 1.0 mm, which were then sealed on the Al_2_O_3_ tube using Ag paste as the sealant. The oxygen permeation fluxes of OPMs were obtained by analyzing the exhaust gas of the OPM-based reactor using gas chromatography. The OPM-based reactor was slowly heated from room temperature (RT) to 900 °C with a rate of 5 °C min^−1^. Argon was used as the sweeping gas at a rate of 50 mL min^−1^. The oxygen permeation test was conducted at 900–600 °C with an interval of 50 °C.

### 2.2. Characterizations

The phase structures of various BCZY samples were acquired by X-ray diffraction (XRD, Rigaku Smart Lab) at the 2θ range of 10–90°. The thermal expansion coefficients (TECs) of the samples were acquired by a dilatometer (DIL 402C, Netzsch, Bayern, Germany) at 300–900 °C in air. The cross-sectional and surface microstructures of BCZY membranes were investigated by scanning electron microscopy (SEM, S-4800, Hitachi, Tokyo, Japan). The thermal properties of various samples in the air were investigated by thermogravimetric analysis (TGA, STA 449 F3, Netzsch, Bayern, Germany). The oxygen non-stoichiometry (δ, oxygen vacancy) values of various perovskite oxides at RT and high temperatures were obtained by titration and TGA, respectively [44]. A mass spectrometer (MS,HPR-20, Hiden Analytical, Warrington, England) was employed to measure the oxygen desorption capability of the samples. The valence states of the samples at RT were acquired by X-ray photoelectron spectroscopy (XPS, AXIS Supra, Shimadzu, Kyoto, Japan). The electrical conductivities of various samples were obtained by a source meter (Keithley 2420, Keithley, Cleveland, OH, America) at 800–500 °C with an interval of 25 °C. The chemical diffusion coefficients (D_chem_) and surface exchange coefficients (K_chem_) of various samples were obtained by electrical conductivity relaxation (ECR) curves tested at 700–500 °C. The ECR responses of various materials were obtained by suddenly switching the oxygen partial pressure from 0.21 to 0.1 atm.

## 3. Results and Discussion

### 3.1. Basic Properties

**Figure 1:**
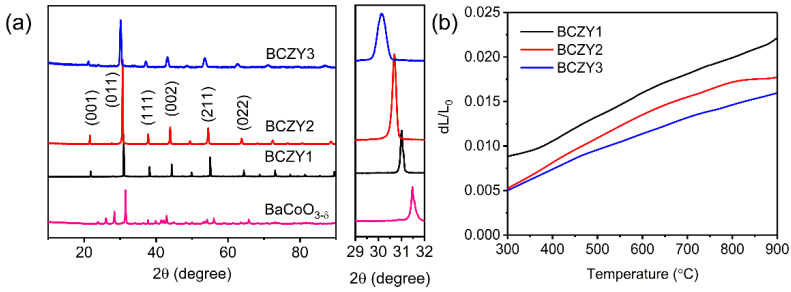
(**a**) RT-XRD patterns and (**b**) Thermal expansion curves of various BCZY samples.

Based on the RT-XRD patterns of various BCZY samples as depicted in Figure 1a, BaCoO_3−δ_ exhibited a hexagonal phase structure while the Zr and Y co-doped BCZY1, BCZY2 and BCZY3 samples showed a pure cubic perovskite structure, indicating that Zr and Y cations were successfully doped into the perovskite lattice without any impurities. Based on the magnified XRD pattern, it was found that the increased co-doping amount led to a shift in diffraction peaks to a smaller angle due to the large cation radius of Zr^4+^ (0.72 Å) and Y^3+^ (0.90 Å) than those of Co^x+^ (0.745, 0.610 and 0.530 Å for Co^2+^, Co^3+^ and Co^4+^). The thermodynamic stability of the cubic structure of BCZY1, BCZY2 and BCZY3 samples were further studied by high-temperature XRD (HT-XRD) with results shown in Figure S1. It was found that BCZY1, BCZY2 and BCZY3 maintained a stable cubic phase structure without any impurity phases at high temperatures, suggesting the high thermal and phase structural stability of Zr and Y co-doped samples, which may be beneficial for the high operation stability of OPMs. As depicted in Figure 1b, BCZY1, BCZY2 and BCZY3 displayed TEC values of 22.2, 20.8 and 18.2 × 10^−6^ K^−1^ at 300–900 °C. It has been reported that the high TEC value of Co-rich perovskites was assigned to the thermal reduction in Co^x+^ cations at high temperatures, leading to a reduced amount of lattice oxygen and increased lattice expansion [45]. The increase in the Zr/Y co-doping amount effectively inhibited the thermal reduction in Co cations and gradually decreased the TEC value of BaCoO_3−δ_, benefiting the achievement of high operational stability of OPMs [46].

**Figure 2:**
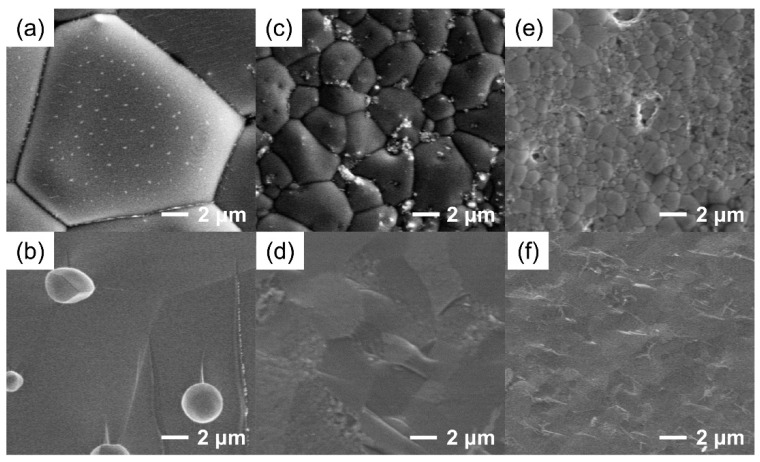
Surface and cross-sectional SEM images of as-prepared BCZY membranes: (**a**,**b**) BCZY1, (**c**,**d**) BCZY2 and (**e**,**f**) BCZY3.

Based on the SEM images of various BCZY membranes as depicted in Figure 2 and Figure S2, clear grain boundaries and no interconnected pinholes were observed, indicating that the dense membranes were obtained. In addition, it was found that BCZY1, BCZY2 and BCZY3 membranes exhibited average grain sizes of 10–30, 3–5 and 1–2 μm, respectively. This indicates that the grain sizes of the as-synthesized perovskite membranes decreased significantly with increased Zr/Y co-doping amounts, which was consistent with Yao et al.’s work where the grain sizes of Zr-doped BaCo_0.7_Fe_0.3_O_3−δ_ decreased with increased Zr doping content [38]. The decrease in grain size of the membranes may be due to the inhibited crystal growth induced by the reduced amount of impurity phase existing at the grain boundary achieved by higher Zr/Y co-doping concentration [47].

### 3.2. Oxygen Permeation Rates

**Figure 3:**
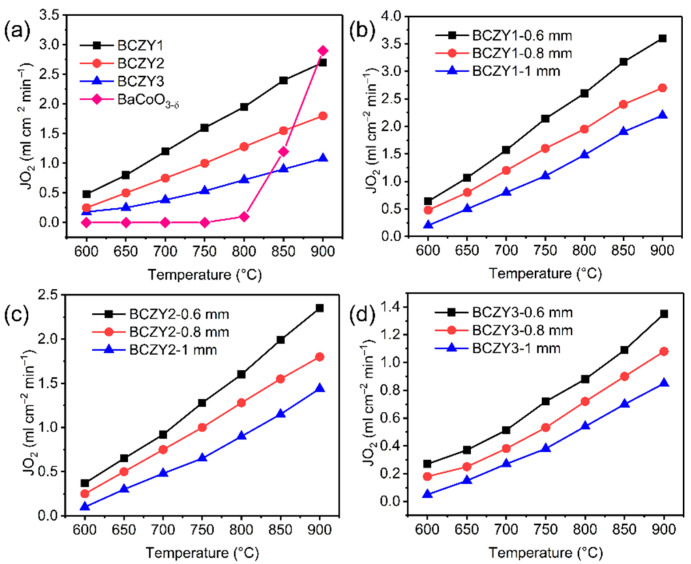
(**a**) Oxygen permeation rates of BaCoO_3−δ_, BCZY1, BCZY2 and BCZY3 membranes with Ar as the sweeping gas at 600–900 °C; Oxygen permeation rates of (**b**) BCZY1, (**c**) BCZY2 and (**d**) BCZY3 membranes with different thicknesses.

Figure 3a shows the oxygen permeation fluxes of various BCZY membranes at different temperatures. As shown in Figure 3a, the oxygen permeation flux obviously increases with the increase in temperature, which is due to the increase in operating temperature is conducive to the bulk phase conduction and surface exchange of oxygen ions of perovskite materials, resulting in higher oxygen permeability [48]. The oxygen permeation rate of BaCoO_3−δ_ at 900 °C was 2.9 mL min^−1^ cm^−2^, which was sharply reduced to only 1.2 mL min^−1^ cm^−2^ at 850 °C due to the detrimental phase transition. Nevertheless, after Zr and Y co-doping, the BCZY membranes displayed much superior oxygen permeability to those of BaCoO_3−δ_ at lower temperatures. The oxygen permeation rates of BCZY1, BCZY2, BCZY3 and BaCoO_3−δ_ were 1.95, 1.28, 0.72 and 0.10 mL min^−1^ cm^−2^ at 800 °C. However, higher Zr/Y co-doping content reduced the oxygen permeation performance of BCZY membranes, which may be caused by the decreased oxygen vacancy concentration. To determine the main factor affecting oxygen permeation of BCZY membranes (e.g., surface exchange or bulk diffusion), the oxygen permeation rates of three BCZY membranes with different thicknesses of 1.0, 0.8 and 0.6 mm were tested at 900–600 °C as depicted in Figure 3b–d. It was found that reducing the thickness of the membrane greatly increased the oxygen permeability and the membrane thickness is nearly inversely proportional to the oxygen permeability (Figure S3). It suggests that the main rate-limiting step of oxygen permeation of BCZY membranes is O^2−^ bulk diffusion and higher oxygen permeability can be achieved by further suppressing the thickness of the membranes [49].

### 3.3. Oxygen Vacancies, Oxidation States and Transport/Diffusion Capability

**Figure 4:**
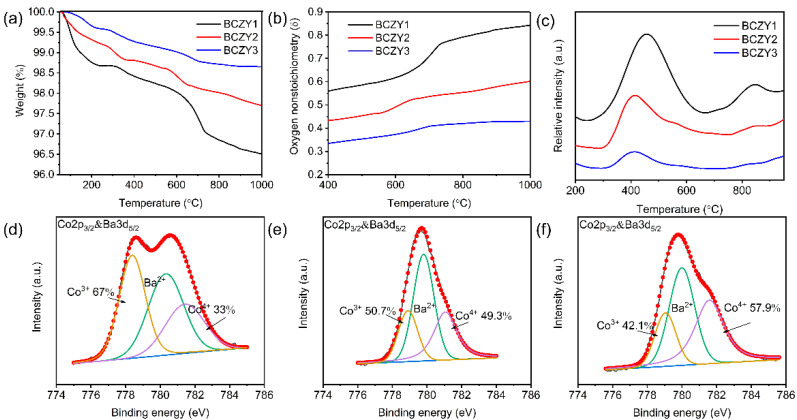
(**a**) TGA curves; (**b**) oxygen vacancy concentration; (**c**) O_2_-TPD curves of BCZY1, BCZY2 and BCZY3; Co 2p and Ba 3d XPS spectra of (**d**) BCZY1, (**e**) BCZY2 and (**f**) BCZY3.

Figure 4a displays the TGA curves of various BCZY samples, demonstrating the weight losses of various co-doped perovskites in the air at 50–1000 °C. It was found that the weight loss of the three samples followed the sequence of BCZY1 > BCZY2 > BCZY3. As shown in Figure 4b, BCZY1 exhibited the largest oxygen vacancy concentration at 400–900 °C while the oxygen vacancy concentration of BCZY3 was much lower than those of the other two samples. It suggested that Zr/Y co-doping inhibited the generation of oxygen vacancies due to the higher valence states of these dopants. A large oxygen vacancy amount favors O^2−^ conduction and improves the oxygen permeability, which may bring a negative effect on the operational stability of OPMs [50]. To further study the effects of Zr/Y co-doping contents on the O^2−^ mobility of various BCZY samples, oxygen temperature programmed desorption (O_2_-TPD) is employed with results shown in Figure 4c. It was found that the initial oxygen desorption temperature of BCZY1, BCZY2 and BCZY3 were 220, 290 and 310 °C, respectively. BCZY1 exhibited the lowest desorption temperature indicating its highest O^2−^ mobility [51]. The Zr/Y co-doping of reduced the O^2−^ mobility, which may contribute to the decreased oxygen permeation rate with increased co-doping amounts. XPS was further conducted to study the influences of Zr/Y co-doping on the oxidation states of B-site Co cations. Based on the Co 2p/Ba 3d XPS spectra and corresponding fitting results of various BCZY samples as depicted in Figure 4d–f and Table S1, the Co^4+^ amounts of BCZY1, BCZY2 and BCZY3 were 33.0%, 49.3% and 57.9% based on the binding energy positions of Co^4+^ and Co^3+^ at 781.0 ± 0.3 and 778.5 ± 0.3 eV in the XPS spectra [52]. The oxygen vacancy amounts of BCZY1, BCZY2 and BCZY3 at RT were calculated to be 0.327, 0.253 and 0.220 based on the XPS results, matching well with the results obtained by titration. These results indicated that the increase in the Zr/Y co-doping amount reduces the oxygen vacancy amount and then the oxygen permeability.

**Figure 5:**
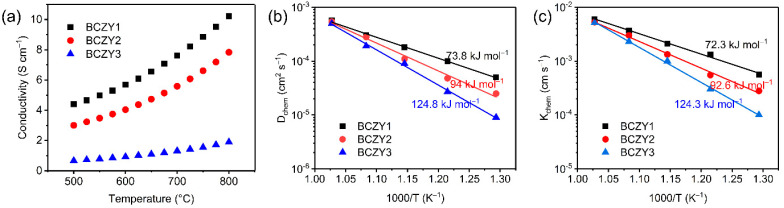
(**a**) Electrical conductivities of BCZY1, BCZY2, BCZY3 in air; Arrhenius plots of (**b**) D_chem_ and (**c**) K_chem_ values of BCZY samples obtained by ECR.

The electrical conductivities of various BCZY samples were tested in air at 500–800 °C as depicted in Figure 5a. Since the ionic conductivity of perovskite oxides is much lower than the electronic conductivity, the electrical conductivity of BCZY samples can be considered electronic conductivity [53,54]. With the increase in the Zr/Y co-doping amount, the electronic conductivities of BCZY were remarkably reduced, especially for the BCZY3 sample. For instance, the electronic conductivities of BCZY1, BCZY2 and BCZY3 samples were 10.2, 7.8 and 1.9 S cm^−1^ in the air at 800 °C, respectively. Replacing Co^x+^ by Zr^4+^ and Y^3+^ cations with fixed valence led to a reduced electrical conductivity, agreeing well with the reported results of Y doped BaCo_0.7_Fe_0.3_O_3−δ_ [55]. To further study the bulk diffusion and surface exchange capabilities of BCZY samples, ECR tests were conducted (Figure S4 and Figure 5b,c and Table S2). It was found that both D_chem_ and K_chem_ values of BCZY samples followed the sequence of BCZY1 > BCZY2 > BCZY3, indicating that the Zr/Y co-doping reduced both the surface exchange and bulk diffusion capability of oxygen species.

### 3.4. Oxygen Permeation Stability, Structural Stability and CO_2_ Tolerance

**Figure 6:**
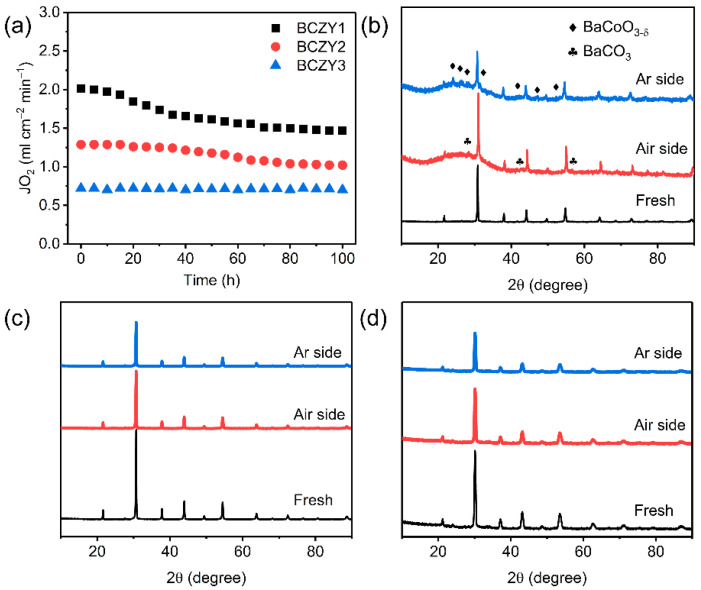
(**a**) Oxygen permeation stability tests of 0.8 mm-thick BCZY1, BCZY2 and BCZY3 membranes at 800 °C; RT-XRD patterns of BCZY membranes after the 100 h’s oxygen permeation: (**b**) BCZY1, (**c**) BCZY2 and (**d**) BCZY3.

Besides the high oxygen permeability, operational stability is also crucial for the OPMs. In this work, the long-term stability of BCZY1, BCZY2 and BCZY3 membranes for oxygen permeation were tested at 800 °C. As depicted in Figure 6a, the oxygen permeation rate of BCZY1 was sharply suppressed from 2.02 to 1.47 mL min^−1^ cm^−2^ after 100 h’s test while BCZY2 and BCZY3 displayed more stable oxygen permeation rates for 100 h. It suggested the Zr/Y co-doping significantly improved the operational stability of the perovskite-based membranes. Furthermore, the phase structures of BCZY1, BCZY2 and BCZY3 membranes after 100 h’s operation were also investigated by XRD as shown in Figure 6b–d. It has been reported that a small amount of carbonate was formed on the surface of BaCoO_3−δ_-based membranes such as BaCo_0.7_Fe_0.2_Sn_0.1_O_3−δ_, which was detrimental to achieving high operational stability of OPMs [56]. As depicted in Figure 6b–d, no obvious changes in the phase structures of BCZY2 and BCZY3 membranes on the air and Ar sides were observed and no carbonates were formed after 100 h’s oxygen permeation test. However, barium carbonate (BaCO_3_) and BaCoO_3−δ_ impurities were observed in the BCZY1 membrane after 100 h’s oxygen permeation, indicating inferior CO_2_ tolerance and structural stability of BCZY1. It can be concluded that the Zr/Y co-doping at higher concentrations is crucial to enhance the CO_2_ resistance of BaCoO_3−δ_-based membranes.

**Figure 7:**
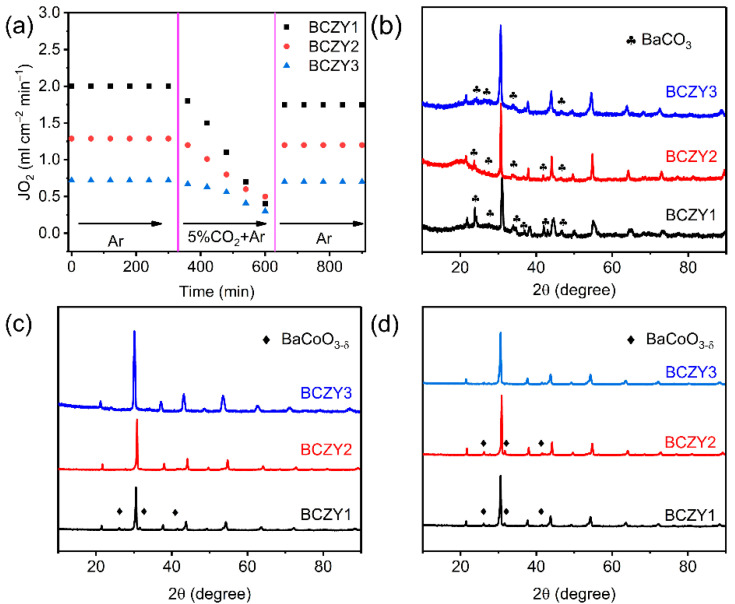
(**a**) Oxygen permeation rates of various BCZY membranes under Ar, CO_2_-Ar and Ar atmospheres at 800 °C; RT-XRD patterns of BCZY1, BCZY2 and BCZY3 samples after a treatment in (**b**) 5%CO_2_ + Ar, (**c**) air and (**d**) O_2_ atmospheres for 20 h at 800 °C.

To further study the CO_2_ tolerance of BCZY-based OPMs, oxygen permeation tests were performed at 800 °C under different sweeping gases in a sequence of pure Ar/5%CO_2_ + Ar/pure Ar for 900 min. As displayed in Figure 7a, the oxygen permeability of all BCZY membranes was reduced when the sweeping gas was changed from Ar to 5%CO_2_ + Ar, which was assigned to the decreased amount of active sites induced by the competitive CO_2_ adsorption and/or the possible carbonate formation on the OPM surface [57,58]. Among them, the BCZY2 membrane delivered the highest oxygen permeation rate of 0.5 mL min^−1^ cm^−2^ after 300 min’s operation in the CO_2_-Ar atmosphere, which was attributed to the improved CO_2_ tolerance and suitable oxygen vacancy concentration [59]. When the sweeping gas was changed back to Ar, the permeation fluxes of BCZY1, BCZY2 and BCZY3 were 1.75, 1.2 and 0.7 mL min^−1^ cm^−2^, respectively. It should be noted that the oxygen permeation rates of BCZY2 and BCZY3 samples were mostly recovered to the primary value due to the higher Zr/Y co-doping concentrations to improve the CO_2_ tolerance of OPMs [60]. Furthermore, BCZY1, BCZY2 and BCZY3 powders were calcined in various atmospheres such as 5%CO_2_ + Ar, air and O_2_ at 800 °C for 20 h to investigate the influences of the treatment atmosphere on the structural stability of perovskite-based OPMs as shown in Figure 7b–d. As shown in Figure 7b, XRD peaks assigned to BaCO_3_ were detected for all three samples after the treatment in 5%CO_2_ + Ar while the increased Zr/Y co-doping amount led to much-reduced peak intensity of such BaCO_3_ impurity, which was assigned to the increased metal-oxygen bonding in perovskite oxides [61]. In addition, the BaCoO_3−δ_ impurity phase was only detected in the BCZY1 sample after the calcination in the air atmosphere for 20 h while BaCoO_3−δ_ minor phase was also observed in BCZY1 and BCZY2 samples after treatment in the O_2_ atmosphere for 20 h (Figure 7c,d). Therefore, we can conclude that the Zr/Y co-doping at higher concentrations significantly improved the phase structural stability of BaCoO_3−δ_-based perovskites towards stable oxygen permeation.

## 4. Conclusions

In summary, the influences of Zr/Y co-doping amounts on the phase structure, grain sizes, electronic conductivity, structural stability, surface exchange/bulk diffusion properties and oxygen permeability of BaCoO_3−δ_ were investigated. With the increased Zr/Y co-doping amount, the electrical conductivity, oxygen vacancy amount and surface exchange/bulk diffusion coefficients of BCZY were gradually decreased and the phase structural stability of co-doped perovskites was improved, leading to gradually reduced oxygen permeability and enhanced oxygen permeation durability. BaCo_0.8_Zr_0.1_Y_0.1_O_3−δ_ with optimized Zr/Y co-doping amount exhibited high permeability and durability for oxygen permeation due to the trade-off between various crucial factors to determine the oxygen permeation performance including grain sizes, crystal structural stability, oxygen vacancy amount and surface exchange/bulk diffusion capability. Thus, BaCo_0.8_Zr_0.1_Y_0.1_O_3−δ_-based OPMs showed a superb oxygen permeation rate of 1.3 mL min^−1^ cm^−2^ and a relatively durable operation for 100 h at 800 °C. This work can provide a high-performance perovskite material for OPMs, which may accelerate the industrialization of OPM technology for oxygen separation.

## Data Availability

The data presented in this study are available on request from the corresponding author.

## Supplementary Materials

The following supporting information can be downloaded at: https://www.mdpi.com/article/10.3390/membranes12090831/s1, Figure S1. HT-XRD patterns of BCZY powder at different temperatures: BCZY1 (a), BCZY2 (b) and BCZY3 (c); Figure S2. Surface SEM image of the BCZY1 membrane; Figure S3. Relationship between oxygen permeation fluxes and the reciprocal thickness of BCZY membranes at different temperatures: (a) BCZY1, (b) BCZY2 and (c) BCZY3; Figure S4. ECR response curves of BCZY obtained between 500 and 700 °C: (a) BCZY1, (b) BCZY2 and (c) BCZY3; Table S1. Average valence states of B-site cations and oxygen vacancy amount of various BCZY samples obtained by XPS and titration; Table S2. The values of D_chem_ and K_chem_ of BCZY samples at various temperatures.
