# Supplementary material for: Zirconium and Yttrium Co-Doped BaCo0.8Zr0.1Y0.1O3−δ: A New Mixed-Conducting Perovskite Oxide-Based Membrane for Efficient and Stable Oxygen Permeation
Source: Membranes (Basel). 2022 Aug 25;12(9):831. doi: 10.3390/membranes12090831 (PMC9501606; doi:10.3390/membranes12090831)
Supplement: Supplementary file 1 [file membranes-12-00831-s001.zip › membranes-1887687-supplementary.pdf]

Supporting Information

Zirconium and Yttrium Co-Doped $BaCo_{0.8}Zr_{0.1}Y_{0.1}O_{3-\delta}$: A New Mixed-Conducting Perovskite Oxide-Based Membrane for Efficient and Stable Oxygen Permeation

Zixiang Xu, Jian Yu*, Wei Wang*

State Key Laboratory of Materials-Oriented Chemical Engineering, College of Chemical Engineering, Nanjing Tech University, Nanjing 210009, China

* Corresponding author.

E-mail address: yuj@njtech.edu.cn (J. Yu), wangwei@njtech.edu.cn (W. Wang)

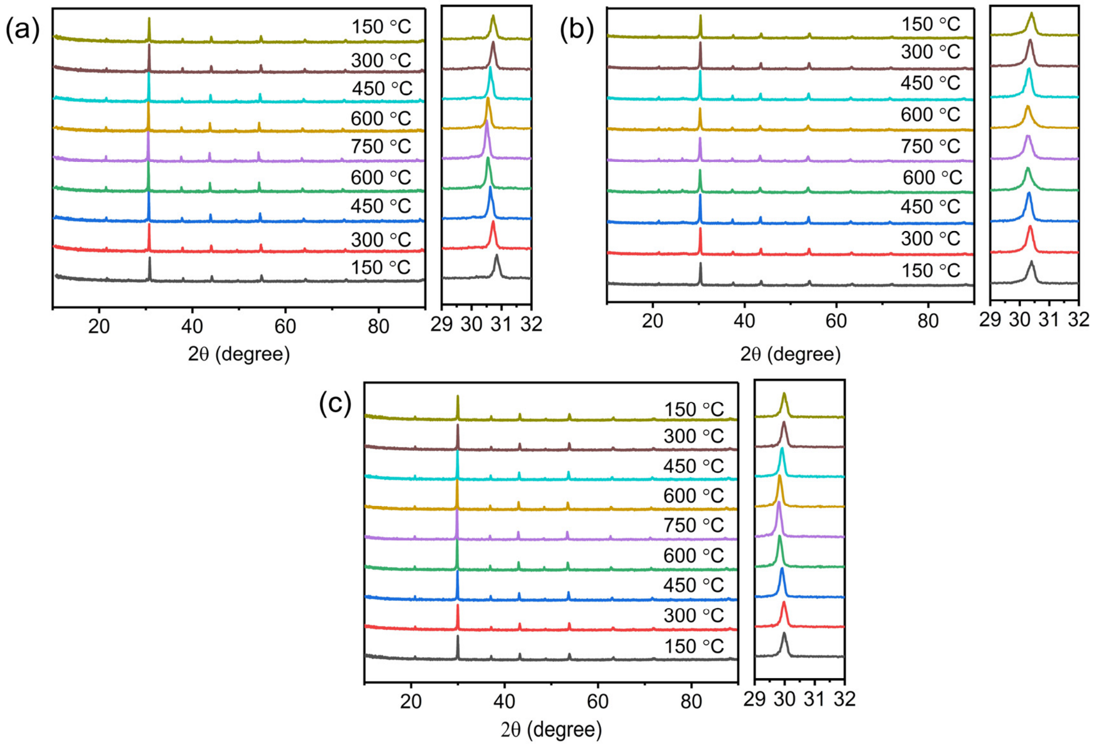

Figure S1. HT-XRD patterns of BCZY powder at different temperatures: BCZY1 (a), BCZY2 (b) and BCZY3 (c).

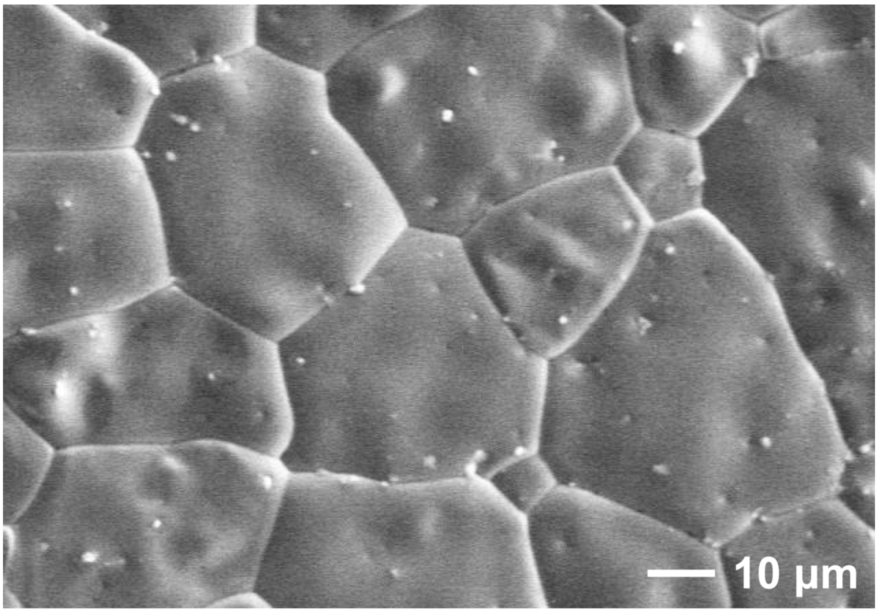

Figure S2. Surface SEM image of the BCZY1 membrane.

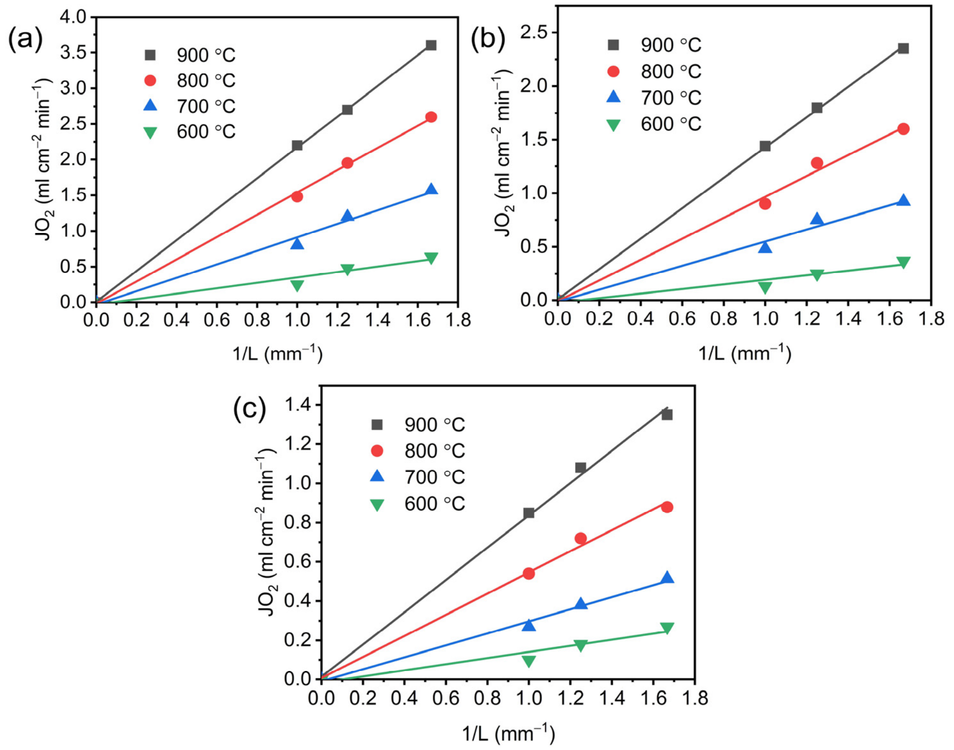

Figure S3. Relationship between oxygen permeation fluxes and the reciprocal thickness of BCZY membranes at different temperatures: (a) BCZY1, (b) BCZY2 and (c) BCZY3.

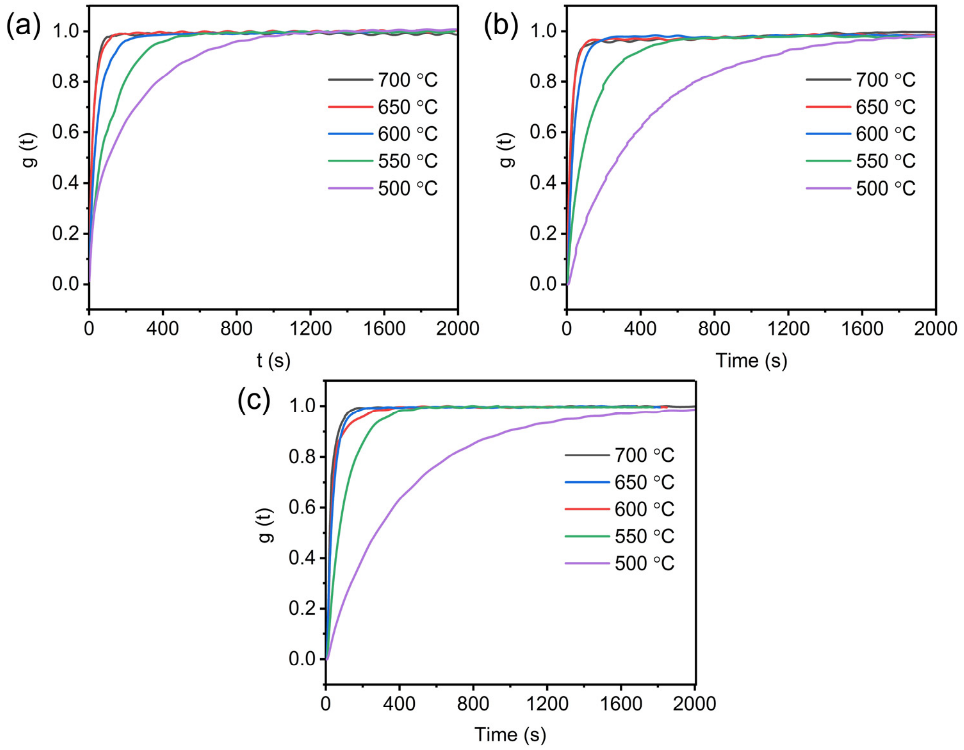

Figure S4. ECR response curves of BCZY obtained between 500 and 700 °C: (a) BCZY1, (b) BCZY2 and (c) BCZY3.

Table S1. Average valence states of B-site cations and oxygen vacancy amount of various BCZY samples obtained by XPS and titration.

Samples	XPS		Titration	
	Co^{3+} (%)	Co^{4+} (%)	3-δ	Average valence states of B-site cations
BCZY1	67.0	33.0	2.66	3.32
BCZY2	50.7	49.3	2.73	3.46
BCZY3	42.1	57.9	2.78	3.56

Table S2. The values of D_{chem} and K_{chem} of BCZY samples at various temperatures.

Sample	Temperature (°C)	700	650	600	550	500
BCZY1	$D*10^4$ (cm^2 s^{-1})	5.65	3.01	1.8	1.00	0.51
	$K*10^3$ (cm s^{-1})	6.00	3.7	2.1	1.33	0.56
BCZY2	$D*10^4$ (cm^2 s^{-1})	5.40	2.8	1.1	0.48	0.25
	$K*10^3$ (cm s^{-1})	5.30	3.00	1.33	0.55	0.28
BCZY3	$D*10^4$ (cm^2 s^{-1})	5.01	1.92	0.90	0.27	0.09
	$K*10^3$ (cm s^{-1})	5.16	2.3	1.00	0.31	0.11
